# IGF-1R nuclear import and recruitment to chromatin involves both alpha and beta subunits

**DOI:** 10.1007/s12672-021-00407-8

**Published:** 2021-04-29

**Authors:** Jack V. Mills, Eliot Osher, Guillaume Rieunier, Ian G. Mills, Valentine M. Macaulay

**Affiliations:** 1 Department of Oncology, University of Oxford, Old Road Campus Research Building, Roosevelt Drive, Oxford, OX3 7DQ UK; 2 Nuffield Department of Surgical Sciences, University of Oxford, Oxford, UK; 3 Oxford Cancer and Haematology Centre, Oxford University Hospitals NHS Foundation Trust, Churchill Hospital, Oxford, OX3 7LJ UK

## Abstract

Mature type 1 insulin-like growth factor receptors (IGF-1Rs) are heterotetrameric structures comprising two extracellular α-subunits disulphide-bonded to two transmembrane β-subunits with tyrosine kinase activity. IGF-1R is a well-known cell surface mediator of malignant growth, with an incompletely understood role upon nuclear import as a transcriptional regulator. Previous characterisation of nuclear IGF-1R focused on IGF-1Rβ. Here, we aimed to clarify the source of nuclear IGF-1R and investigate whether α-subunits contribute to nuclear IGF-1R function. Using prostate cancer cell lines DU145 and 22Rv1 we detected nuclear α- and β-subunits, with increase in nuclear signal upon IGF-treatment and reduction in response to IGF-1R inhibitor BMS-754807. Following biotinylation of cell surface proteins, biotinylated α- and β-subunits were detected in nuclear extracts of both cell lines. Furthermore, α- and β-subunits reciprocally co-precipitated from nuclear extract. Finally, we detected recruitment of both subunits to regulatory regions of chromatin, including the promoter of the oncogene *JUN*, that we previously identified in ChIP-seq as sites of IGF-1Rβ enrichment. These data confirm the cell surface origin of nuclear IGF-1R, suggest the presence of nuclear αβ complexes and reveal that both IGF-1Rα- and β-subunits contribute to pro-tumorigenic functions of nuclear IGF-1R.

**Supplementary Information:**

The online version contains supplementary material available at 10.1007/s12672-021-00407-8.

## Introduction

**Fig. 1:**
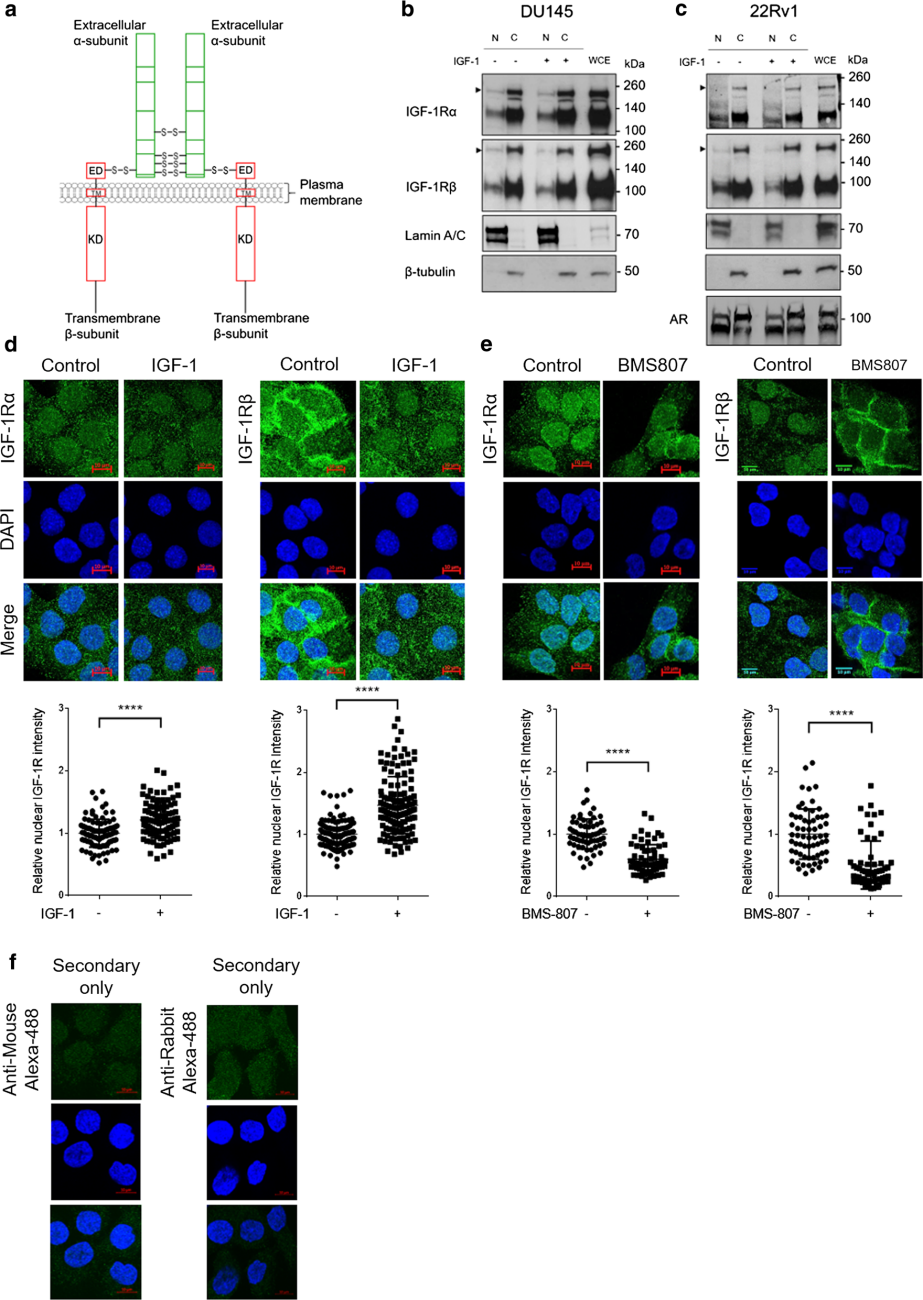
Full length IGF-1Rα- and β-subunits are detectable in the nucleus of prostate cancer cells **a** Domain structure of mature IGF-1R at the plasma membrane, showing extracellular α-subunit (green), transmembrane β-subunit (red), extracellular (ED), transmembrane (TM), kinase domains (KD) and disulphide bonds, modified from [3]. **b**, **c** Western blots showing detection of both IGF-1Rα- and β-subunits in the nucleus of DU145 (**b**) and 22Rv1 (**c**) cells. Cells were serum-starved for 24 h, treated with solvent (4.5 µM HCl) or IGF-1 (50 nM) for 20 min before fractionation. All western blots shown are representative of 3 independent biological repeats. Arrowheads: 220 kDa IGF-1R proreceptor. **d** Images showing immunofluorescence staining of IGF-1Rα and IGF-1Rβ in DU145 cells. DU145 cells were serum-starved and IGF-treated as in b-c followed by immunofluorescent staining. Graphs: mean ± SD nuclear IGF-1Rα- and β-subunits (n ≥ 30 cells per condition for n = 3 biological repeats). Data were analysed by unpaired student’s t test and quantification of nuclear levels of IGF-1Rα- and β-subunits showed a significant increase on treatment with IGF-1 (*p < 0.05; **p < 0.005; ***p < 0.001; ****p < 0.0001) **e** Images showing IGF-1Rα and IGF-1Rβ immunofluorescence in DU145 cells. Cells were grown in full medium with 10% FCS and incubated with solvent (0.1% DMSO) or IGF-1R inhibitor BMS-754807 (100 nM) for 6 h at 37 °C and processed for immunofluorescence staining. Graphs: mean ± SD nuclear IGF-1Rα- and β-subunits (n ≥ 30 cells per condition for n = 2 biological repeats). Data were analysed by unpaired student’s t test and quantification of nuclear levels of IGF-1Rα- and β-subunits showed a significant decrease on treatment with BMS-754807 (*p < 0.05; **p < 0.005; ***p < 0.001; ****p < 0.0001) **f** Images showing cultured DU145 cells stained with Alexa-488 secondary antibodies as used in **d** and **e** in the absence of primary antibody. These images represent a negative control for immunofluorescence staining and demonstrate the specificity of the IGF-1Rα- and β-subunit signals shown in **d** and **e**

Type 1 insulin-like growth factor receptor (IGF-1R) is a ubiquitously expressed, transmembrane receptor tyrosine kinase (RTK), closely related to the insulin receptor (INSR) and the main mediator of IGF related signalling and activity [1–3]. Like INSR, IGF-1Rs are translated as ~ 220 kDa pro-receptors containing signal peptide and α- and β-subunit sequence that are co-translationally inserted into the endoplasmic reticulum (ER). After disulphide-bonding and folding, IGF-1Rs are transported to the trans-Golgi network where proreceptor cleavage generates α-subunits (~ 140 kDa) and β-subunits (~ 100 kDa) [3], forming heterotetrameric holoreceptors in a disulphide-bonded α_2_β_2_-configuration. These mature receptors undergo trafficking to the plasma membrane, where the extracellular α-subunits form a complex binding site for a single IGF molecule and the β-subunits, which contain small extracellular and transmembrane domains, transmit signals via the intracellular tyrosine kinase domain [4] (Fig. 1a). Under normal physiological conditions IGF axis signalling is tightly regulated, however the IGF axis has long been recognised for its contribution to cancer growth and metastasis by promoting cell survival, proliferation and invasion [5, 6]. Because of these functions the IGF axis is a target for therapeutic intervention [7]. Despite some cases showing exceptional responses, the majority of trials involving IGF-1R inhibitors have failed, due to factors including lack of predictive biomarkers, dose-limiting hyperglycaemia resulting from co-inhibition of INSR, and incomplete understanding of IGF-1R biology [8–10].

Work from our group and others revealed that upon activation, IGF-1R, like many other RTKs, can translocate to the nucleus, the initial trafficking step involving clathrin mediated-endocytosis [11–14]. We also reported that nuclear IGF-1R undergoes ligand-induced recruitment to regulatory regions of chromatin, and interacts with RNA Polymerase II (RNAPolII) to promote RNAPolII recruitment and expression of genes including *JUN* that drive tumour cell proliferation and migration [15]. Others find that nuclear IGF-1R also interacts with transcription factors and nuclear proteins to alter their function [16–18].

Data from Larsson and colleagues indicate that IGF-1R translocation requires SUMOylation of 3 conserved β-subunit lysine residues followed by an importin-β/RANBP2-meditated nuclear import mechanism [12, 14]. We found that nuclear IGF-1R is more frequently detected in malignant than benign epithelium, and is associated with advanced tumour stage in prostate cancer and reduced overall survival in renal cancer [11, 15]. There is also evidence that nuclear IGF-1R associates with response to IGF-1R antibody therapy in patients with sarcoma [19], suggesting that nuclear IGF-1R may indicate dependence on the IGF axis.

The majority of this work to elucidate nuclear IGF-1R function has focussed on detection of the β-subunit only, so it is unclear whether the receptor α-subunit also undergoes translocation and whether it is involved in the nuclear function of IGF-1R. While our lab and others were previously able to detect full-length IGF-1Rα- and β-subunits in the nucleus by subcellular fractionation and immunohistochemistry [11, 12, 20], it is unclear to what extent the α-subunit contributes to the nuclear function of IGF-1R. Here, the objective was to determine whether there is a functional role of the IGF-1Rα-subunit in the nucleus, distinct or otherwise from the β-subunit, aiming to further elucidate the biology of nuclear IGF-1R and understand its contribution to cancer biology as well as its potential as a therapeutic target.

In this study we used two prostate cancer cell lines, DU145 and 22Rv1, that are respectively androgen receptor (AR) negative and positive, a receptor whose function is critical for prostate cancer progression [21, 22] and with which there is known cross-talk with the IGF-axis [23, 24]. We used subcellular fractionation to detect both α- and β-subunits in the nucleus of both DU145 and 22Rv1 prostate cancer cells and showed by immunofluorescence microscopy that the α-subunit of nuclear IGF-1R is responsive to IGF-1R inhibition, analogous to previous results for the β-subunit [11]. Using cell surface protein biotinylation, we confirmed that both IGF-1R subunits detected in the nucleus had originated from the cell surface, confirming a widely accepted hypothesis in the field. We also showed, via co-immunoprecipitation from nuclear extract, that the two nuclear subunits interact with each other, suggesting that IGF-1R may undergo translocation as holoreceptors or αβ half receptors. We also detected recruitment of the IGF-1Rα-subunit to previously identified IGF-1Rβ-subunit chromatin binding sites, suggesting that IGF-1Rα-subunit is also involved in the nuclear function of the receptor.

## Methods

### Cell lines

DU145 (from Cancer Research UK Clare Hall Laboratories) and 22Rv1 prostate cancer cells (obtained from Professor Sir Walter Bodmer, University of Oxford) were cultured in RPMI 1640 medium with 10% fetal calf serum (FCS). Both were mycoplasma-free when tested with MycoAlert (Lonza Rockland Inc.). DU145 and 22Rv1 cells were authenticated by STR genotyping in 2016 (by Eurofins Medigenomix Forensik GmbH and Cancer Research UK Clare Hall Laboratories respectively). After confirming cell line identity, early passage stocks were expanded and cryopreserved, and used within 20 passages of recovery.

### Western blotting and antibodies

Cell fractions and whole cell extracts (WCE, prepared in IGF-1R lysis buffer as described in [11]) were resolved by SDS-PAGE and western blotting using antibody for IGF-1Rα [Santa Cruz Biotech. (Heidelberg, Germany), sc-712], IGF-1Rβ [Cell Signalling Technology (Leiden, The Netherlands), CST-3027], EGFR (D38B1) XP (Cell Signalling Technology, CST-4267), AR (Cell Signalling Technology, CST-5153), Lamin A/C (BD Biosciences (Wokingham, UK) 612162) and β-tubulin (Cell Signalling Technology, CST-86298).

### Subcellular fractionation

Cells were fractionated into nuclear (N) and cytoplasmic (C) fractions using NE-PER™ Nuclear and Cytoplasmic Extraction Reagent kit (ThermoFisher (Paisley, United Kingdom) Cat. No. 78833) as per manufacturers protocol with minor modifications. Briefly, following appropriate treatment, cells were collected by cell scraping in PBS, pelleted by centrifugation at 1200 rpm, 5 min at 4 °C and incubated for 15 min on ice in Cytoplasmic Extraction Reagent (CER) I following 1 min vigorous vortexing. Following addition of CER II, samples were vortexed vigorously for 20 s and incubated on ice for a further 3 min. Samples were again vortexed vigorously for 20 s before centrifugation at 15 000 rpm, 5 min at 4 °C. Supernatants (cytoplasmic extract) were removed and stored at − 80 °C until analysis. Pelleted nuclei were washed once in ice cold PBS, before addition of Nuclear Extraction Reagent (NER) and incubation for 1 h at 4 °C with agitation. Following incubation samples underwent centrifugation at 15,000 rpm, 10 min at 4 °C and supernatant (nuclear extract) was removed and stored at − 80 °C until analysis.

### Cell surface protein biotinylation

Cells were serum-starved overnight and incubated for 10 min with PBS control or EZ-Link™ Sulfo-NHS-SS-Biotin label using the Pierce™ Cell Surface Biotinylation and Isolation Kit protocol (ThermoFisher Cat. No. A44390) according to the manufacturer’s protocol. Cells were then washed twice with ambient PBS, treated with RPMI-1640 medium supplemented with 10% FCS for 20 min at 37 °C and then fractionated into nuclear (N) and cytoplasmic (C) fractions as described previously. Biotinylated proteins were eluted using NeutrAvadin Agarose spin columns and analysed by western blot alongside N, C and WCE input controls.

### Co-immunoprecipitation

Cells were cultured in RPMI-1640 medium with 10% FCS and fractionated into N and C fractions as above. Equal concentrations of nuclear lysates were precleared by incubation with Protein A agarose beads [Merck Millipore (Watford, UK) Cat. No. P9269] and Normal Rabbit IgG antibody (CST-2729) for 1 h at 4 °C with rotation. Precleared nuclear extracts were immunoprecipitated overnight at 4 °C with rotation with antibody for IGF-1Rα (ThermoFisher (24–60, MA5-13817), IGF-1Rβ (CST-3027) or Normal Rabbit IgG control (CST-2729). Immunoprecipitated proteins were collected by 2 h incubation at 4 °C with rotation with Protein A agarose beads (Merck Millipore (Watford, UK) Cat. No. P9269) and analysed alongside N, C and WCE input controls by western blotting.

### Immunofluorescence staining and microscopy

Immunofluorescence staining was performed as per Cell Signalling Technology protocol. Briefly, cells were fixed with 4% paraformaldehyde [methanol free, Alfa Aesar (Heysham, UK) 43368) in ambient PBS for 20 min at room temperature (RT), incubated in Blocking Solution [5% Bovine Serum Albumin (BSA) and 0.3% Triton X-100 in ambient PBS] for 1 h at room temperature. Blocking Solution was removed and cells were incubated overnight at 4 °C with primary antibodies diluted in Blocking Solution: IGF-1Rα (24–60, MA5-13817, ThermoFisher, 1:250) or IGF-1Rβ (CST 9750, 1:500). After washing, cells were incubated in secondary antibody diluted in blocking solution: goat anti-mouse IgG (H+L, Highly Cross-Adsorbed Secondary Antibody, Alexa Fluor 488, 1:2000, Invitrogen, ThermoFisher) for cells incubated with primary antibody for the α-subunit, or goat anti-Rabbit IgG (H+L Highly Cross-Adsorbed Secondary Antibody, Alexa Fluor 488 1:2000, Invitrogen, ThermoFisher) for cells incubated with primary antibody for the β-subunit, for 1 h at room temperature with protection from light. Cover slips were mounted using mounting medium containing 4′,6-diamidino-2-phenylindole (DAPI). Slides were stored at 4 °C until visualisation on a Zeiss LSM 780 Confocal microscope (Carl Zeiss, Germany) using ×63 magnification objective. Alexa Fluor 488 was excited with laser line 488 nm and emission collected between 480 and 670 nm. Alexa Fluor 594 was excited with laser line 594 nm and emission collected between 590 and 745 nm. Nuclear IGF-1R was assessed using FIJI (ImageJ) software by quantifying IGF-1R signal within the boundaries of outlined DAPI stained nuclei.

### Chromatin immunoprecipitation and qPCR

Cultured cells were fixed with 1% formaldehyde before being processed using Merck Millipore ChIP kit (Cat. No. 17-295) as per the manufacturers protocol, with minor modifications as in [15]. Chromatin was pre-cleared using salmon sperm DNA/Protein A agarose slurry (Millipore ChIP kit, Cat. No. 17-295) with Normal Rabbit IgG (CST-2729). Samples were immunoprecipitated using antibody for IGF-1Rα (ThermoFisher, 24-60, MA5-13817) or IGF-1Rβ (CST-3027). Immunoprecipitated proteins were collected using salmon sperm DNA/Protein A agarose slurry (Millipore ChIP kit, Cat. No. 17-295) and processed as per Merck Millipore ChIP kit protocol to generate DNA samples. The qPCR was performed using Luna® Universal qPCR Master Mix (New England Biolabs (Hitchin, UK) Cat. No. M3003) as per the manufacturers protocol using primer sequences for *JUN* promoter and an intergenic region of chromosome 17 (IntChr17) described in [15].

## Results and discussion

### Both IGF-1Rα- and β-subunits are detectable in the nucleus of prostate cancer cells

DU145 and 22Rv1 prostate cancer cell lines showed detectable levels of nuclear IGF-1Rα- and IGF-1Rβ-subunits in nuclear extracts prepared from cells cultured in both serum-starved and IGF-treated conditions (Fig. 1b, c). There was no evidence of significant change in nuclear IGF-1R content upon IGF-1 stimulation, possibly reflecting non-linearity of western blotting. We previously used immunofluorescence to assess IGF-induced changes in IGF-1R subcellular localisation for both subunits, but tested the response of only nuclear IGF-1Rβ-subunit to IGF-1R kinase inhibition [11]. Here, we again assessed the localisation of both subunits in serum-starved DU145 cells in the presence or absence of IGF-1. Immunofluorescence confirmed detection of IGF-1R in the nucleus with evidence that both IGF-1Rα- and IGF-1Rβ-subunits were IGF-1 responsive, showing a significant increase upon IGF-1 treatment (Fig. 1d), as previously shown [11]. In addition, we found evidence of significant IGF-1R membrane retention on treatment with IGF-1R tyrosine kinase inhibitor BMS-754807, with reduction in nuclear IGF-1Rα-subunit and accumulation of IGF-1R on the plasma membrane (Fig. 1e) as previously seen with the IGF-1Rβ-subunit [11] and also shown here (Fig. 1e). These data confirm our previous findings with respect to IGF-1Rβ-subunit translocation in DU145 cells [11], show that both IGF-1Rα- and β-subunits are detectable in the nucleus of 22Rv1 cells, and provide additional evidence that the α-subunit also undergoes IGF-induced nuclear translocation.

### Nuclear IGF-1R undergoes translocation from the cell surface

We next wanted to check whether IGF-1R in the nucleus had originated from the cell surface. It is widely assumed that IGF-1R undergoes ligand stimulation at the cell surface before translocation to the nucleus [11–15], but this has never been directly tested, although we have previously shown a reduction in nuclear IGF-1Rβ-subunit in DU145 cells in response to inhibition of clathrin-mediated endocytosis [11]. Given that the outer nuclear membrane is in continuity with the ER membrane, it has been proposed that some nuclear proteins could undergo nuclear import via direct lateral diffusion, the rapid process of lateral movement of proteins within a membrane [25].

To assess the route of IGF-1R trafficking, DU145 and 22Rv1 cells underwent biotinylation of cell surface proteins followed by ligand stimulation and subcellular fractionation. Biotinylated proteins were isolated from nuclear extract and probed by Western blot for presence of IGF-1Rα- and β-subunits. The biotinylation labelling reagent is membrane impermeable and labels proteins via formation of an amide bond between the primary amine of lysine residues and the biotin label [26]. The IGF-1Rα-subunit is completely extracellular [1, 3] and contains multiple primary amine residues that we determined would be labelled in the presence of the external biotinylation reagent. The IGF-1Rβ-subunit is almost completely intracellular, although does contain a small extracellular domain (shown in Fig. 1a) that contains multiple lysine residues within the fibronectin type III (FnIII)-2b and -3 domains at Lys-782, -821, -838, -857 and -897 [ref. 1–4]. Therefore, both subunits were predicted to undergo biotinylation.

**Fig. 2:**
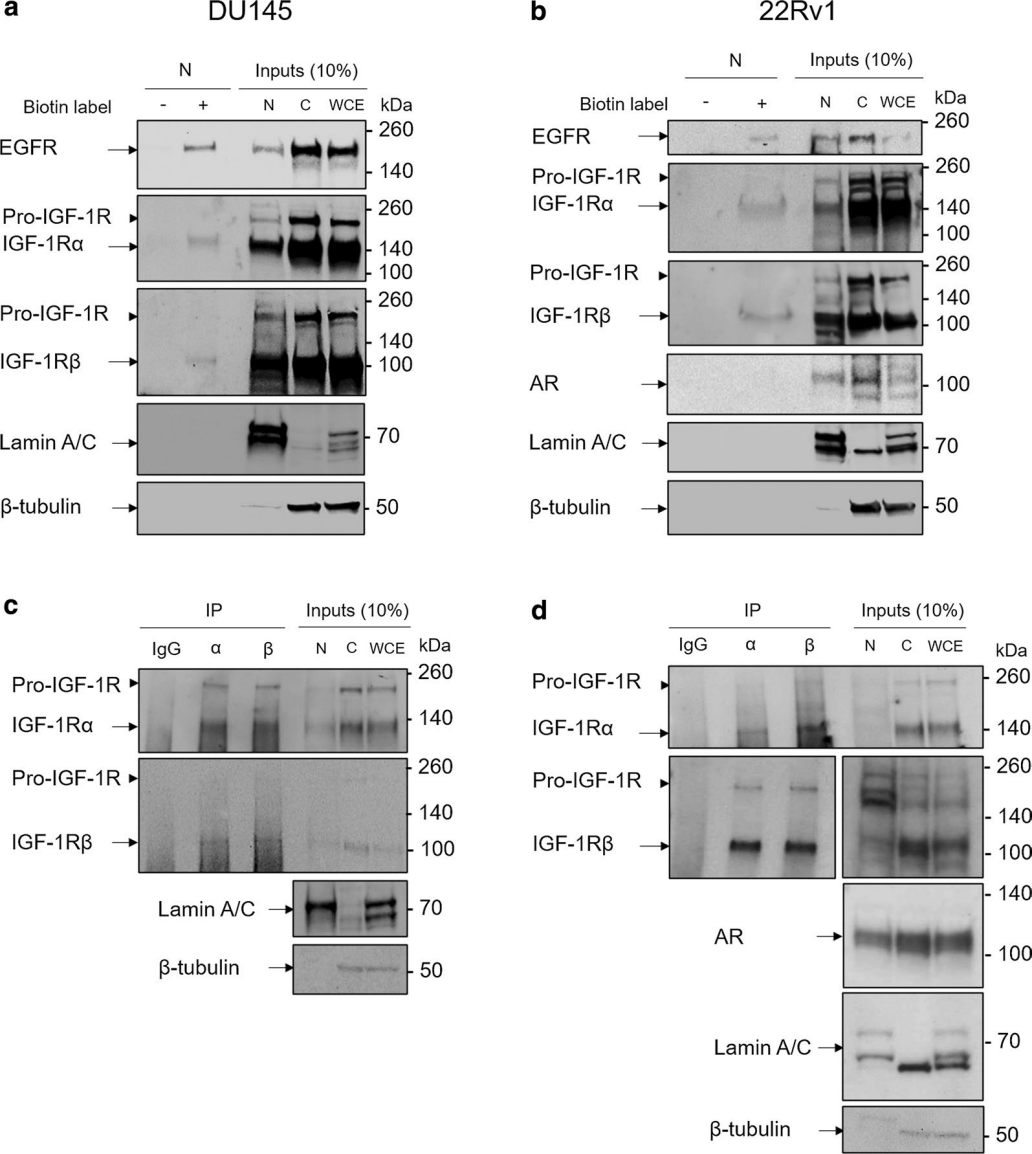
Nuclear IGF-1Rα- and β-subunits originate from the cell surface and interact in the nucleus **a, b** Western blots showing detection of cell surface biotinylated IGF-1R in the nucleus of DU145 (**a**) and 22Rv1 (**b**) cells. Detection of cell surface biotinylated EGFR in nuclear fractions acts as a positive control. Western blots shown are representative of 3 independent biological repeats. **c, d** Co-immunoprecipitation Western blot showing interaction of IGF-1Rα- and β-subunits in the nucleus of DU145 (**c**) and 22Rv1 (**d**) cells. Western blots shown are representative of 3 independent biological repeats

As expected, both subunits could be detected in nuclear, cytoplasmic and whole cell extract (WCE) protein input controls in both cell lines. Furthermore, both the α- and β-subunits were detectable in nuclear extracts from cells that had undergone cell surface protein biotinylation. Neither subunit was detected in control extracts that had undergone incubation with PBS rather than biotinylation reagent (Fig. 2a, b). As a positive control, nuclear extracts were also shown to contain cell surface biotinylated epidermal growth factor receptor (EGFR), a protein well characterised to translocate from the cell surface to the nucleus [27–29]. In contrast, there was no detection of biotinylated lamin and, in 22Rv1, AR, which are both nuclear proteins that do not traffic from the cell surface. Given that only cell surface proteins had been exposed to biotinylation, the absence of both IGF-1R subunits in control samples and presence in nuclear extracts from surface biotin-labelled cells confirms that IGF-1R subunits detectable in the nucleus have translocated from the cell surface.

### IGF-1Rα- and β-subunits interact in the nucleus

Following confirmation that nuclear IGF-1R had translocated from the cell surface, we next wanted to investigate whether the two subunits of the receptor retain their interaction in the nucleus. Following cellular fractionation of DU145 and 22Rv1 cells, α- or β-subunits were immunoprecipitated from nuclear extracts. As before, both subunits were detected in the nuclear, cytoplasmic and WCE protein input controls. Following immunoprecipitation from nuclear extract, the α-subunit of IGF-1R could be detected in both DU145 and 22Rv1 immunoprecipitates. The IGF-1Rβ-subunit could also clearly be detected following co-immunoprecipitation using α-subunit antibody. Reciprocal β-subunit immunoprecipitation also detected both IGF-1Rα- and β-subunits in DU145 and 22Rv1 nuclear extracts (Fig. 2c, d).

These experiments indicated interaction of IGF-1Rα- and β-subunits in the nucleus of prostate cancer cells. This finding could suggest that IGF-1R translocates as an α_2_β_2_ holoreceptor. The majority of RTKs that undergo nuclear import, including fibroblast growth factor and EGFR family members, translocate after juxta-membrane proteolytic release of cytoplasmic domain or nuclear import of full-length receptor monomers [27–29]. In contrast, IGF-1R and the closely related INSR are heterotetramers in which the α_2_β_2_ structure is maintained by disulphide bonds [3]. Nuclear INSR was reported originally by [30] and recently by Hancock et al. [31], the latter finding INSR interaction with RNAPolII, consistent with our data for IGF-1R [15], and showing by immunogold electron microscopy that a subset of nuclear INSRα- and β-chains were sufficiently close to each other for direct interaction [31]. Our finding of α–β co-precipitation implies retention of disulphide-bonded subunits during trafficking from the endosomal compartment via the cytosol to the nucleus. The cytosol is a mildly reducing environment in which most thiols are in a reduced state but which can maintain disulphide-bonds in proteins transferred from other cellular compartments [32]. IGF-1R and INSR contain similar numbers of alpha and beta cysteines; mild INSR reduction is known to cleave a small number of disulphides, generating disulphide-linked αβ dimers that can still bind ligand, while more vigorous INSR reduction generates free alpha and beta chains [3]. Our detection of IGF-1R α-β complexes in nuclear extract implies existence of similar IGF-1R αβ dimers or α_2_β_2_ holoreceptors.

### IGF-1Rα is recruited to IGF-1Rβ DNA binding sites

**Fig. 3:**
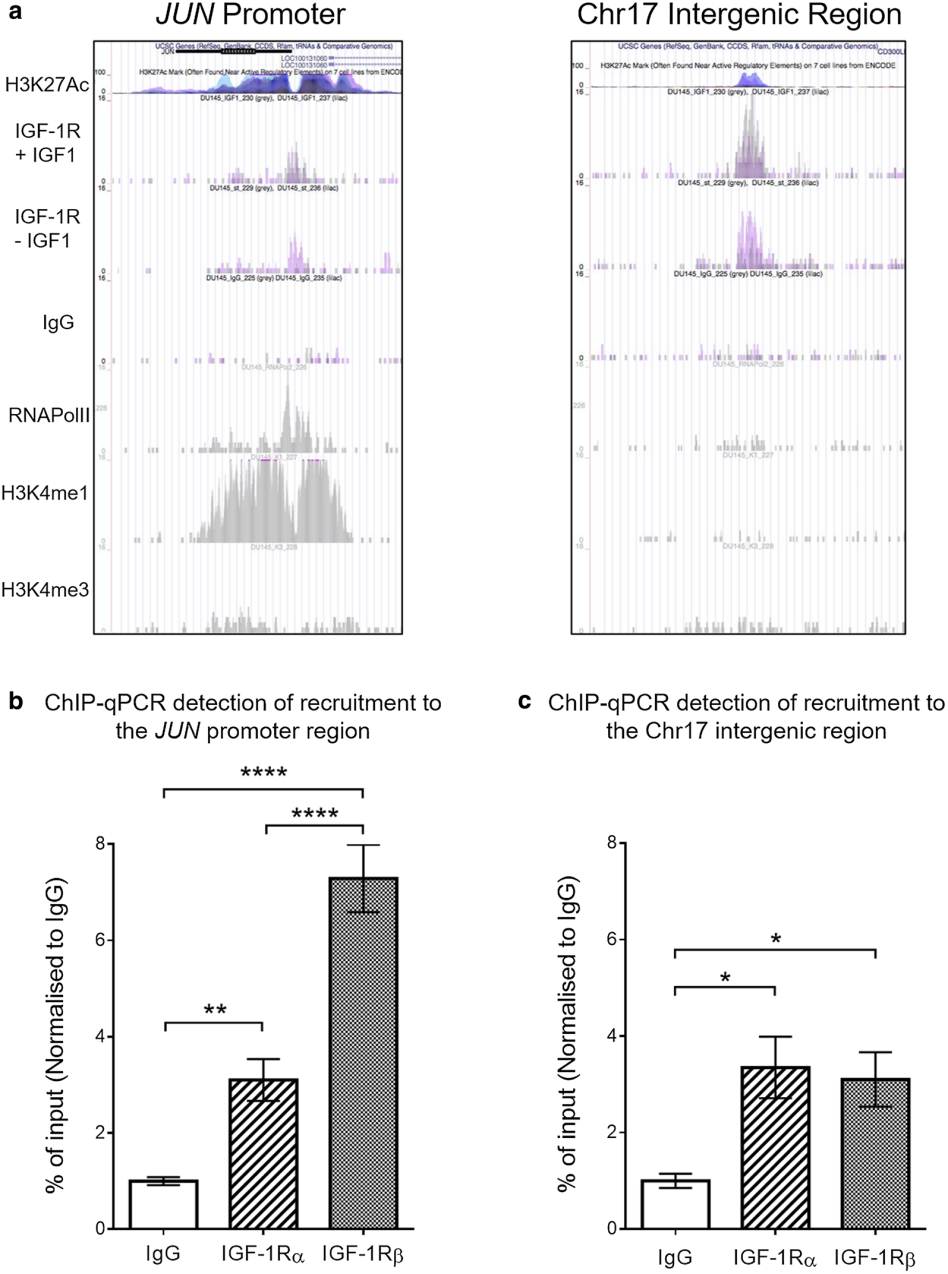
Recruitment of IGF-1Rα is detectable at IGF-1Rβ DNA binding sites. **a** Images from the UCSC genome browser of previously obtained ChIP-seq data showing IGF-1R binding peaks that denote recruitment of RNAPolII and IGF-1Rβ to the *JUN* promoter and IntChr17 [ref 15]. **b**, **c** Quantification of recruitment of IGF-1Rα or IGF-1Rβ vs IgG negative control to IGF-1Rβ binding regions, detected by ChIP-qPCR. Results represent mean ± SEM of triplicate independent experiments, each with 3 technical replicates. Recruitment of both IGF-1Rα and IGF-1Rβ vs IgG was detected at the *JUN* promoter (**b**) and putative IntChr17 enhancer **(c;** p* < 0.05, p** < 0.005, p**** < 0.0001, Tukey's multiple comparisons test)

Our previous ChIP-seq in DU145 cells revealed IGF-1Rβ recruitment to regulatory regions of chromatin. At most of these sites, IGF-1Rβ enrichment was coincident with recruitment of RNAPolII, and direct interaction between IGF-1Rβ and RNAPolII was also detected. Both IGF-1Rβ recruitment and RNAPolII interaction were shown to be IGF-dependent [15]. Here, having detected nuclear IGF-1Rα-β complexes (Fig. 2c, d), we investigated whether IGF-1R α-subunit contributes to nuclear IGF-1R function. Therefore, we tested for IGF-1Rα recruitment to two regions previously validated as sites of IGF-1Rβ enrichment [15]. One is the promoter of the *JUN* oncogene which we confirmed to contribute to prostate cancer cell growth and motility [15], and the other an intergenic region of chromosome 17 (IntChr17) that is enriched for H3K27 acetylation but not RNAPolII enrichment (Fig. 3a), suggesting putative enhancer function [33]**.**

As previously shown by our group [15], ChIP-qPCR in DU145 cells was able to detect significant recruitment of IGF-1Rβ to the *JUN* promoter (Fig. 3b), and here we also showed recruitment to IntChr17 (Fig. 3c), validating the ChIP-seq finding. At the *JUN* promoter region both subunits showed significant increase in recruitment versus IgG negative control, however α-subunit recruitment was found to be at a significantly lower level than that of the β-subunit (Fig. 3b). Although not shown here, to increase confidence in the specificity of this result, it would be helpful in future work to include ChIP-qPCR for an unrelated genomic region not previously found to be a site of IGF-1Rβ recruitment. At the IntChr17 binding site, both subunits showed significant increase in recruitment at approximately equal levels (Fig. 3c). These results suggest that the IGF-1R α-subunit is also involved in recruitment of IGF-1R to chromatin. This mirrors the closely related INSR, which shares significant sequence homology, similar structure and overlapping functions with IGF-1R [1, 3, 34, 35], where recent work reported similar levels of recruitment of both α- and β- INSR subunits to INSR chromatin binding sites validated by ChIP-qPCR [31].

It is worth noting that RNAPolII is not recruited to the IntChr17 region tested here, in contrast to the RNAPolII recruitment seen at the *JUN* promoter region [15] (Fig. 3a). As previously mentioned, our lab has shown direct interaction of RNAPolII and IGF-1R [15], therefore it is possible that this interaction is disrupting immunoprecipitation and detection of the α-subunit at the *JUN* promoter region, resulting in differing levels of detected recruitment for the α- and β-subunits at this site. These differences could also be due to technical factors such as different efficiency of antibodies for immunoprecipitation from chromatin, masking of DNA-interacting domain by antibody binding, and/or interaction of the α-subunit indirectly via the β-subunit.

**Fig. 4:**
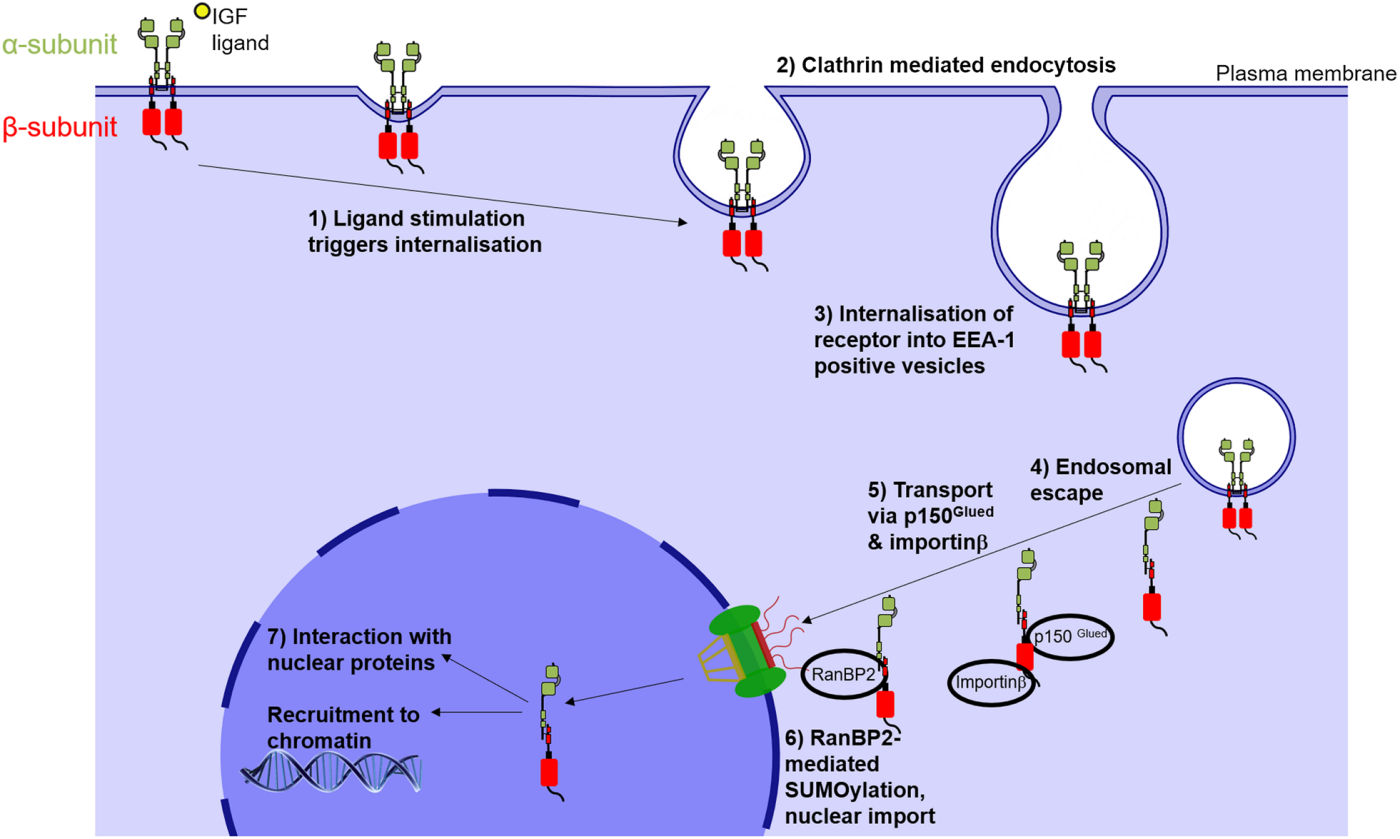
Model of potential nuclear translocation mechanism of IGF-1R in its holoreceptor form **1–3**. Ligand stimulation of IGF-1R triggers clathrin mediated endocytosis of the receptor into EEA-1 positive vesicles [11, 14]. **4**. IGF-1R is presumed to undergo endosomal escape into the cytosol. Detection of nuclear IGF-1Rα-β complexes in this manuscript suggests retention of disulphide bonded-subunits during transit through the cytosol. **5–6**. Cytosolic IGF-1R interacts with p150^Glued^ and is transported to the nucleus via microtubule-dependent dynactin activity where it interacts with importin-β followed by RanBP2. RanBP2 E3 SUMO ligase catalyses the tri-SUMOylation of 3 conserved lysine residues of the β-subunit [12, 14]. **6.** SUMOylation mediates IGF-1R translocation across the nuclear pore complex and into the nucleus. **7.** Nuclear IGF-1R interacts with nuclear proteins including PCNA, nucleolar protein NOM1, transcriptional regulators including TCF/LEF1, Histone H3, RNAPolII, GATA-2 and with regulatory regions of chromatin to influence gene transcription and protein function [15–18, 36]

Taken together, we have shown that both IGF-1Rα- and β-subunits are detectable in the nucleus of 22Rv1 prostate cancer cells, building on previous results seen in DU145 cells [11]. Cell surface protein biotinylation experiments directly confirmed for the first time that IGF-1Rs detectable in the nucleus originate from the cell membrane, in line with a model in which the receptor undergoes translocation to the nucleus following ligand stimulation at the cell surface. We have also shown by co-immunoprecipitation that α- and β-subunits form nuclear complexes, suggesting that the receptor translocates as an α-β dimer or holoreceptor (Fig. 4). Finally, using ChIP-qPCR we detected α-subunit recruitment to a region of the *JUN* promoter and an intergenic region of Chromosome 17, previously identified as IGF-1Rβ binding sites [15]. These findings suggest that IGF-1Rα-subunit is involved in IGF-1R chromatin binding and transcriptional activation of genes including *JUN* that act as prostate cancer oncogenes, therefore contributing towards the more advanced tumour stage in prostate cancer and reduced overall survival in clear cell renal cancer associated with nuclear IGF-1R [11, 15].

While we have not directly assessed the prognostic value of nuclear IGF-1R signalling in this work, as mentioned above, we have previously found an association between nuclear IGF-1R and advanced tumour stage in patients undergoing radical prostatectomy. We also showed a correlation between nuclear IGF-1Rβ-subunit and JUN in malignant prostate epithelium by immunohistochemistry [15]. However, as the patients from this cohort were generally a good prognostic group, it was not possible to assess association of nuclear IGF-1R with prostate cancer survival. To test the association of nuclear IGF-1R with prognosis and survival would require further work involving immunohistochemistry on large numbers of cases supported by good clinical data. Such datasets could also be used to test the correlation of *JUN* expression with IGF-1R activation via staining for phospho-IGF-1R. Future work is also needed to determine what further nuclear IGF-1R chromatin binding sites and protein interactions can now be detected via the α-subunit that may further elucidate the role of nuclear IGF-1R in cancer progression.

## Supplementary Information

Below is the link to the electronic supplementary material.

Supplementary file1 (XLSX 86 KB)

## Data Availability

The data in this manuscript was generated from original work, and any and all raw data files can be made available on request. The IGF-1R ChIP-seq data set analysed to identify IGF-1R chromatin binding sites is available from the corresponding author upon reasonable request.
